# The craniocervical junction: embryology, anatomy, biomechanics and imaging in blunt trauma

**DOI:** 10.1007/s13244-016-0530-5

**Published:** 2016-11-04

**Authors:** Curtis Edward Offiah, Emily Day

**Affiliations:** Department of Neuroradiology, Imaging Department, Royal London Hospital, Barts Health NHS Trust, Whitechapel, London, E1 1BB UK

**Keywords:** Trauma, Nervous system, Spinal cord injuries, Ligaments, Fractures

## Abstract

**Abstract:**

Imaging of the blunt traumatic injuries to the craniocervical junction can be challenging but central to improving morbidity and mortality related to such injury. The radiologist has a significant part to play in the appropriate management of patients who have suffered injury to this vital junction between the cranium and the spine. Knowledge of the embryology and normal anatomy as well as normal variant appearances avoids inappropriate investigations in these trauma patients. Osseous injury can be subtle while representing important radiological red flags for significant underlying ligamentous injury. An understanding of bony and ligamentous injury patterns can also give some idea of the biomechanics and degree of force required to inflict such trauma. This will assist greatly in predicting risk for other critical injuries related to vital neighbouring structures such as vasculature, brain stem, cranial nerves and spinal cord. The embryology and anatomy of the craniocervical junction will be outlined in this review and the relevant osseous and ligamentous injuries which can arise as a result of blunt trauma to this site described together. Appropriate secondary radiological imaging considerations related to potential complications of such trauma will also be discussed.

***Teaching points*:**

• *The craniocervical junction is a distinct osseo-ligamentous entity with specific functional demands*.

• *Understanding the embryology of the craniocervical junction may prevent erroneous radiological interpretation*.

• *In blunt trauma, the anatomical biomechanical demands of the ligaments warrant consideration*.

• *Dedicated MRI sequences can provide accurate evaluation of ligamentous integrity and injury*.

• *Injury of the craniocervical junction carries risk of blunt traumatic cerebrovascular injury*.

## Introduction

The craniocervical (craniovertebral) junction represents the complex transitional zone between the cranium and the spine and comprises a complex balance of different elements: it should be considered anatomically and radiologically a distinct entity from both the cranium and, in particular, the cervical spine. It is composed of osseous structures articulated with synovial joints, intrinsic ligaments and membranes and muscles. As well as housing the spinal cord and multiple cranial nerves, it is also approximated by critical vasculature supplying both the brain and the cervical spinal cord parenchyma. As a result, injury to the craniocervical junction carries the potential for devastating morbidity and mortality. The requirements placed on the craniocervical junction are onerous—not only must it house, protect and support structures critical for function (and ultimately evolutionary survival), it must also simultaneously provide significant mobility.

In the setting of blunt trauma to the craniocervical junction, imaging plays an indispensable role in the management and prognostication of these injuries. The acute imaging evaluation of what are usually high-energy mechanisms of injury typically involves an initial computed tomography (CT) assessment and, frequently, subsequent emergency magnetic resonance imaging (MRI) assessment. The role of these imaging modalities and the biomechanics and appearances of typical injuries of the craniocervical junction will be reviewed. For consideration of blunt trauma affecting the sub-axial cervical spine (i.e. trauma of the cervical spine below the craniocervical junction), the reader is directed to other such dedicated reviews [1].

## Embryology

**Fig. 1:**
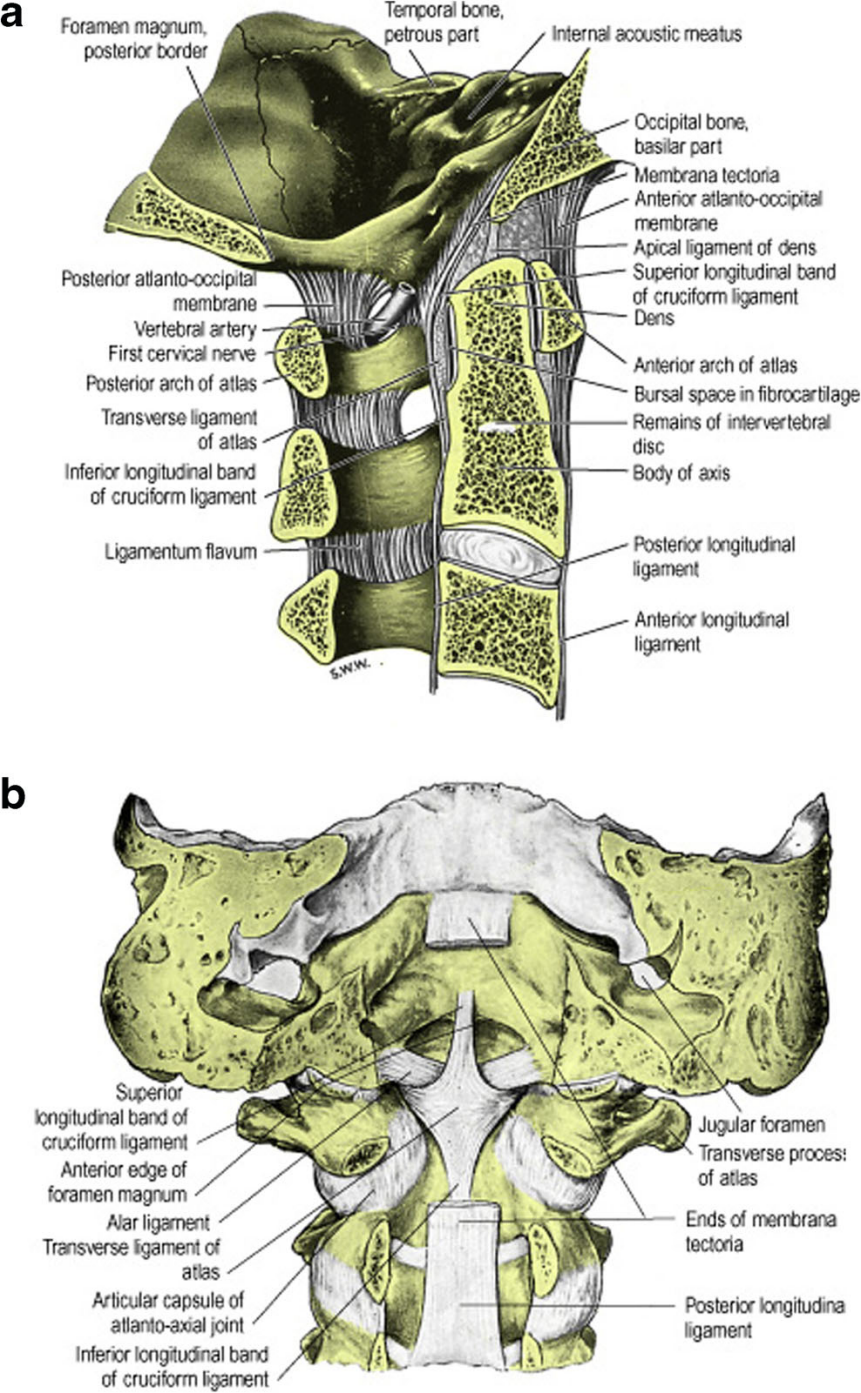
Illustrative anatomy of the craniocervical junction osseo-ligamentous structures: **a** right lateral view of sagittally sectioned craniocervical junction in a median plane (i.e. viewed from right to left); **b** posterior view of the coronally sectioned craniocervical junction; the tectorial membrane has been partly removed to expose deeper ligaments. (These illustrations were published in Gray’s Anatomy: The Anatomical Basis of Clinical Practice (39th Edition), Susan Strandring, The Back, Copyright Elsevier, 2005)

In relation to the biomechanics of the craniocervical junction and the impact of trauma on this structure, it is useful to visualise the craniocervical junction as composed of two components: the first is a central pillar consisting of the central basiocciput (even though it is anatomically part of the skull base), odontoid process (dens or peg) and the C2 vertebral body; the second component consists of the two-ringed structures surrounding the central pillar—these are the ring of the foramen magnum including the lateral portions of the basiocciput, the exocciput incorporating the occipital condyles and the opisthion (the posterior margin of the foramen magnum), and the ring of the C1 vertebra (atlas) consisting of the anterior and posterior arches and lateral masses of the atlas [2–8] (Fig. 1). Functionally, the latter stacked two-ringed component allows limited rotation around the central pillar as well as intrinsic limited flexion-extension. Ligaments bind these two structural components of the craniocervical junction providing stability. Knowledge of the embryology of the craniocervical junction is germane to this analogy of a two-component structure and also helps to confidently distinguish developmental anomalous/normal variant appearances of the imaged osseous craniocervical junction from genuine traumatic injury. While detailed description of the embryological development of the craniocervical junction is beyond the scope of this review, a brief summary of selected aspects will be given.

The craniocervical junction is of mesodermal origin which appears in the 3rd gestational week. During gastrulation, cells from the embryonic plate condense to form the parachordal mesoderm on each side of the notochord. This mesoderm eventually separates into segmental clusters called somites. These paired somites (the number of which is species-specific—42 pairs in humans) will eventually give rise to the smooth muscle of the dermis, axial skeletal musculature and the vertebral column (amongst other structures). Once mature, somites differentiate into ventromedial sclerotomes and dorsolateral dermomyotomes. The sclerotomes eventually develop into vertebral bodies, neural arches, ligaments and membranes [2–8].

**Fig. 2:**
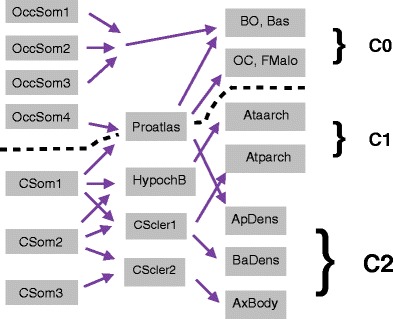
Schematic of craniocervical junction developmental segmentation and resegmentation during embryogenesis. Abbreviations: OccSom1 - occipital somite 1; OccSom2 - occipital somite 2; OccSom3 - occipital somite 3; OccSom4 - occipital somite 4; CSom1 - cervical somite 1; CSom2 - cervical somite 2; CSom3 - cervical somite 3; CScler1 - cervical sclerotome 1; CScler2 - cervical sclerotome 2; BO - basiocciput; Bas - basion; OC - occipital condyles; FMalp - anterolateral foramen magnum and opisthion; Ataarch - anterior arch of C1 vertebra (atlas); Atparch - posterior arch of C1 vertebra (atlas); ApDens - apical segment of the dens; BaDens - basal segment of the dens; body of C2 vertebra (axis). Severed black line denotes the embryological separation between the cranial skull base and cervical spine during craniocervical junction development

The craniocervical junction develops from the four occipital somites and the first three cervical somites (Fig. 2). The first three occipital somites will give rise to the rostral basiocciput. The fourth occipital somite combines with the cranial part of the first cervical somite to form the sclerotome called the proatlas which is the precursor of the craniocervical junction. The cranial part of the axial region of this sclerotome fuses with sclerotome segments of the first three occipital somites to form the basion of the basiocciput. The caudal part of the axial region of the proatlas derived from the first cervical somite gives rise to the apical segment of the dens. The lateral region of the proatlas eventually gives rise to the occipital condyles and the remainder of the anterolateral foramen magnum [3, 4, 6] (Fig. 2).

The caudal half of somite five and the cranial half of somite six combine to form the first cervical sclerotome. Similarly, the caudal half of somite six and the cranial half of somite seven combine to form the second cervical sclerotome. Part of the first cervical sclerotome gives rise to the basal part of the odontoid peg (dens) while part of the second cervical sclerotome gives rise to the body of the axis (C2 vertebra). A distinct feature of the first and second cervical sclerotomes compared to the more caudal sclerotomes is the absence of the conversion of part of the sclerotomes to the annulus fibrosus and nucleus pulposus of the intervertebral discs. Instead, this tissue disappears and the mesenchyme at these junctional sites turns into the upper dental synchondrosis between the apical dens and basal dens and the lower dental synchondrosis between the basal dens and the body of the axis. Hence, the C2 vertebra is distinctive in that it is derived from three adjacent sclerotomes which ultimately give rise to cranial as well as vertebral elements and this vertebra is the true embryological juncture of the cranium and the spine and, therefore, must, somewhat challengingly, serve the biomechanical requirements of both [3, 6].

### The atlas and the axis embryology

The lateral zone of the first cervical sclerotome develops into the posterior arch of the C1 vertebra while the lateral zone of the second cervical sclerotome develops into the arch of the axis (C2 vertebra; Fig. 2). The anterior arch of the C1 vertebra develops from a small mesenchymal off-shoot ventral to both the notochord and the axial segment of first cervical sclerotome called the hypochordal bow. [The hypochordal bow of the proatlas gives rise to a small osseous midline tubercle attached to the ventral surface of the basiocciput below the anterior margin of the foramen magnum (basion) and frequently visible on CT (and MRI) of the mature normal craniocervical junction].

### Ligament embryology

The apical ligament is derived from the axial proatlas. The alar ligaments and the transverse ligamentous component of the cruciform (cruciate) ligament develop from the axial component of the first cervical sclerotome.

### Chondrification and ossification of C2 vertebra and radiology

**Fig. 3:**
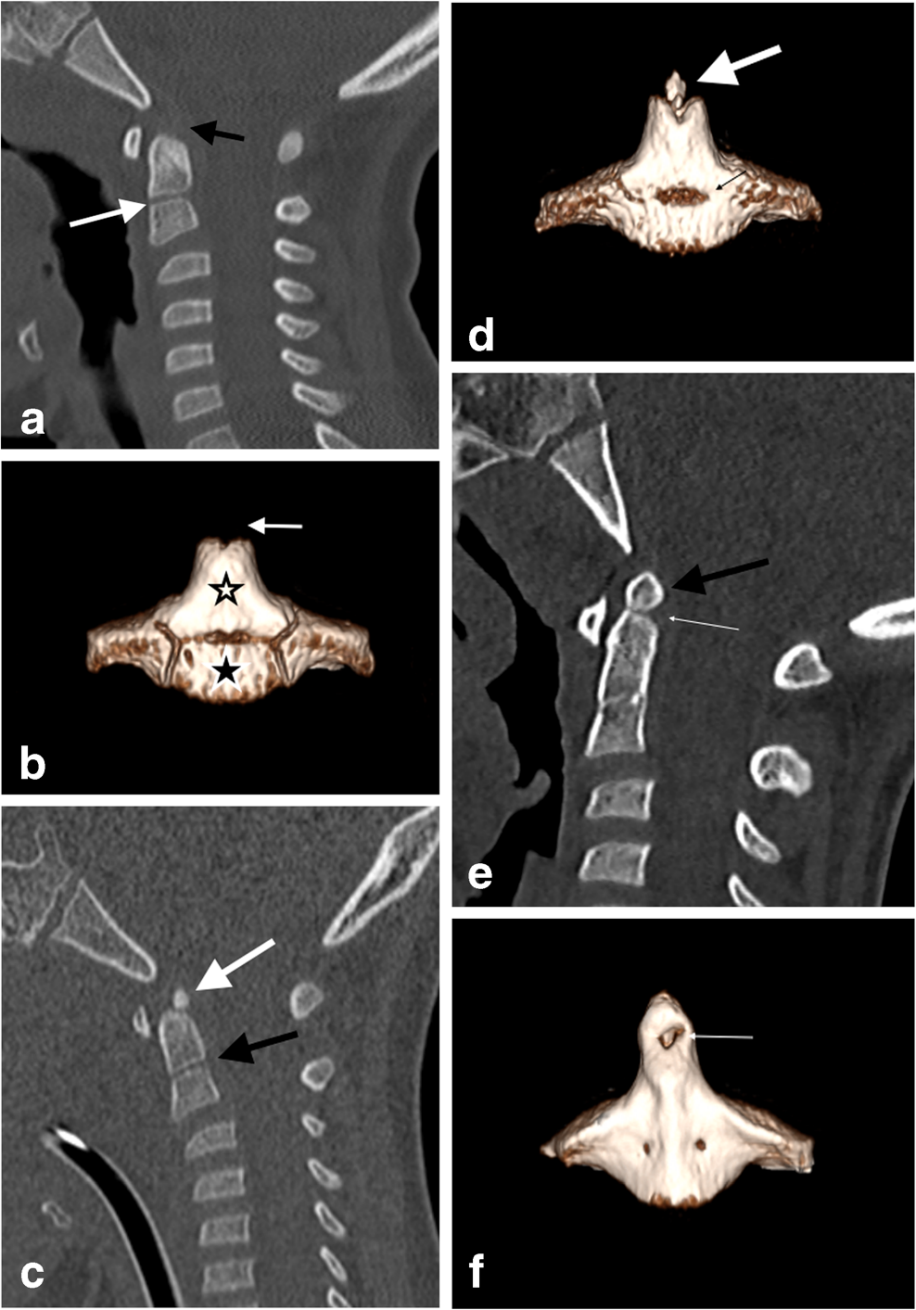
Progressive ossification of C2 vertebra: **a**: sagittal reconstructed CT image of the craniocervical junction of a 6-year-old male who fell 30 feet off a balcony—ossification has not yet begun in the chondrified apical dens (*black arrow*) in this 6-year old and the lower dental synchondrosis (*white arrow*) is also evident as non-ossified cartilage. C2-C3 pseudosubluxation is present.; **b**: volume-rendered three-dimensional reconstructed CT image of the C2 vertebra of the same 6-year-old male—the apical dens is non-ossified (*arrow*) and the ossified basal dental segment (*outlined asterisk*) and the body of the C2 vertebra (*black asterisk*) have not yet fused; **c**: sagittal reconstructed CT image of the craniocervical junction of a 5-year-old male who fell from a first floor window—ossification has begun but is incomplete in the chondrified apical dental segment (*white arrow*) and a thin ossifying lower dental synchondrosis (*black arrow*) is evident. The appearances should not be misinterpreted as fracture changes despite the high-energy clinical information apparent. C2-C3 pseudosubluxation is again present; **d**: volume-rendered three-dimensional reconstructed CT image of the C2 vertebra of the same 5-year-old male—the ossification of the apical dens is evolving (*white arrow*) and the lower dental synchondrosis is still visible (*thin black arrow*); **f**: sagittal reconstructed CT image of the craniocervical junction of a 6-year-old female who was a pedestrian struck by a car travelling at moderate speed—advanced ossification of the apical dental segment is present (*black arrow*) with early ossification across the upper synchondrosis (*white arrow*). Pseudosubluxation at the C2-C3 level is present; **f**: volume-rendered three-dimensional reconstructed CT image of the C2 vertebra of the same 6-year-old female—ossification across the upper synchondrosis (*thin white arrow*) and lower synchondrosis exhibits progressive features as union of the ossification centres proceeds

The membranous C2 vertebra in the early part of the first trimester consists of three median segments: the apical dental segment, the basal dental segment and the body of the axis. These three segments chondrify at 6 weeks of gestation but remain segregated by the upper and lower dental synchondroses [8–11]. Ossification occurs in three stages: the first ossification centre appears in the body of the the C2 vertebra at 4 months of gestation; the second wave of ossification appears at 6 months of gestation as two ossification centres, one on each side of the basal dental segment—these two centres integrate and fuse at birth when they should have begun to show some attempt at osseous fusion but the non-ossified cartilage may still be evident radiologically into the 6th year of life (Fig. 3a–f); the third wave of ossification appears in the apical dens at 3 to 5 years of age—ossification of the apical dens and fusion across the upper dental synchondrosis is not complete until adolescence (Fig. 3a–f) [8–11].

## Anatomy and biomechanics

**Fig. 4:**
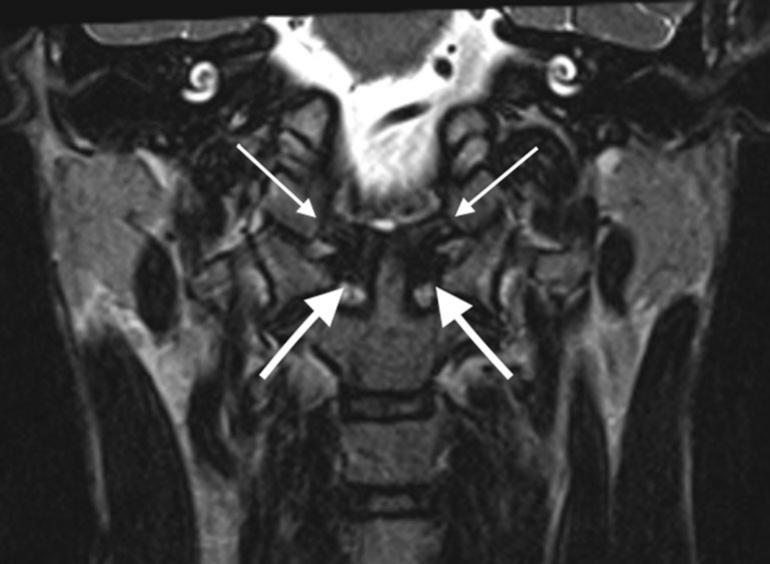
Coronal three-dimensional T2 SPACE (sampling perfection with application-optimised contrasts using different flip-angle evolutions) sequence image through normal craniocervical junction [Siemens 1.5 Tesla MRI scanner: slice thickness: 0.8 mm (no interslice gap), TR (time to repetition): 1500 ms, TE (time to echo): 129 ms, FOV (field of view): 160 mm, number of excitations (NEX) averages: 1.6, matrix: 261 × 256, acquisition time: 6.5 ]in) demonstrating normal MRI appearances of the transverse ligament (*thick arrows*) and alar ligaments (*thin arrows*)

The craniocervical junction is composed of two major joints: the atlanto-occipital joint and the atlanto-axial joint (Figs. 1 and 4). These two joints are responsible for the majority of the movement available in the entire cervical spine and the anatomical structure of each is based on different biomechanical principles. The mechanical properties of the atlanto-occipital joint are primarily determined by bony structures, whereas those of the atlanto-axial joint are primarily determined by ligamentous structures.

### The occipital bone and the atlanto-occipital (C0-C1) joints

The occipital bone encompasses the foramen magnum and extends from the clivus anteriorly to the lambdoid suture posteriorly. The occipital condyles angle medially and inferiorly from the posterior to anterior: this angulation limits the mobility of the atlanto-occipital joints (i.e. C0-C1 joints), particularly in axial rotation compared with the atlanto-axial joint (i.e. C1-C2 joint) [12–14]. The predominant movements at the atlanto-occipital joint are flexion and extension. Lateral flexion at the atlanto-occipital joint is significantly limited by the contralateral alar ligament.

### The atlanto-axial (C1-C2) joints

The atlanto-axial joints (i.e. C1-C2 joints) allow mobility in flexion, extension, axial rotation and, to a lesser degree, lateral flexion as a result of the biconvex and inherently unstable construct of the joint; it is the ligaments (transverse ligament and alar ligaments) related to this particular articulation which stabilise the joint complex. In the event of traumatic disruption of these ligaments, the atlanto-axial joints are poorly equipped to tolerate axial rotation. This is in stark contrast to the atlanto-occipital joints which are less affected by ligamentous injury [15, 16].

## Craniocervical ligaments

### Alar ligaments

These paired ligaments attach the axis to the base of the skull (Figs. 1 and 4) and originate from the posterior surface of the upper third of the dens and typically travel caudocranially (in 50 % of cadeveric dissections) or horizontally (in 50 % of subjects); the exact insertion point of the alar ligaments has been subject to some contention with researchers variably describing insertion on the medial aspect of the occipital condyles or the anterolateral aspect of the foramen magnum [14, 17–21]. Each ligament is narrowest at its origin and comparatively wider at its insertion giving it a “V-shaped” configuration [20]. The alar ligaments limit axial rotation and lateral flexion on the contralateral side and, apart from the transverse ligament, are the strongest stabilisers of the atlas preventing anterior displacement in the event of rupture of the transverse ligament. Together with the transverse ligament (transverse atlantal ligament; see below), the alar ligaments are primary stabilisers of the craniocervical junction.

### Cruciform ligament (cruciate ligament)

The cruciform ligament is composed of transverse and vertical parts which form a cross behind the odontoid peg (Figs. 1 and 4) [14, 16, 19]. The vertical component which is relatively weak and offers no discernible craniocervical stability consists of a cranially orientated longitudinal band which inserts on to the upper surface of the clivus between the apical ligament and tectorial membrane and a caudally directed band which inserts on to the posterior surface of the body of the axis.

### Transverse ligament

The transverse ligament (sometimes termed the transverse atlantal ligament) of the cruciform ligament complex is arguably the most important ligament in the body. It is the largest, thickest and, crucially, the strongest of the craniocervical junction ligaments (and, in fact, the strongest ligament in the entire spine) and, therefore, a primary stabiliser of the craniocervical junction. It arches behind the odontoid peg attaching to a tubercle arising from the medial aspect of each lateral mass of the atlas (Figs. 1 and 4). The transverse ligament is central to stability of the craniocervical junction, fixing the odontoid peg firmly to the posterior aspect of the anterior arch of the atlas. A synovial capsule is situated between the odontoid process and the transverse ligament. The tectorial membrane, epidural fat and dura are located posterior to the transverse ligament. The transverse ligament serves as the major stabiliser of the atlanto-axial articulation: it permits rotation at the atlanto-axial joints while, at the same time, the alar ligaments will prevent excessive rotation. Tears of the transverse ligament typically occur laterally at the attachment to the tubercle on the atlas.

### Tectorial membrane

This thin structure represents an upward extension of the posterior longitudinal ligament (Fig. 1). It forms the posterior border to the supraodontoid space or apical “cave” [14, 22] and runs posterior to the cruciform ligament. It extends cranially to the clivus (as far cranially as the spheno-occipital synchondrosis) and caudally to the posterior surface of the body of the axis. It attaches as far laterally as the hypoglossal canals and, at the level of C0-C1, merges with the atlanto-occipital capsular ligaments (Arnold’s ligaments). The cranial portion of the membrane is adherent to and anatomically indistinguishable from dura [14, 23].

### Capsular ligaments

The capsular ligaments of the atlanto-occipital and atlanto-axial joints (which are paired synovial joints) are typically described as thin and loose.

### Apical ligament

This ligament extends from the tip of the odontoid process to the basion and is situated between the anterior atlanto-occipital membrane and the cruciform ligament (Fig. 1); it is surrounded by fat, connective tissue and a venous plexus which accounts for the slightly variable signal characteristics of this supraodontoid space or “apical cave” on MR imaging. It may be absent in up to 20 % of subjects based on cadaveric dissections undertaken by Tubbs et al. [14, 19, 22, 24]. Despite it being renowned, this ligament probably offers little, if any, significant contribution to craniocervical junction stability.

### Anterior atlanto-occipital membrane

This thin structure attaches the anterior aspect of the atlas to the anterior rim of the foramen magnum (Fig. 1) and is located immediately posterior to the prevertebral muscles [14, 19, 25]. It forms the anterior wall of the supraodontoid space (which is very discernible on MRI assessment owing to its contents of fat and veins) which also houses the alar and apical (and Barkow) ligaments. It serves to limit atlanto-occipital extension at the craniocervical junction.

### Posterior atlanto-ocipital membrane

Although believed to play very little part in stability of the atlantooccipital articulation, the posterior atlanto-occipital membrane is important because it is highly visible on MRI assessment and also exhibits some specific anatomical features which may be misinterpreted on imaging as traumatic disruption. This broad ligament attaches the posterior arch of the atlas to the posterior margin of the foramen magnum and is continuous with the posterior atlantoaxial membrane and, subsequently, the ligamentum flavum [14, 19, 25] (Fig. 1). Laterally, it may extend over the capsules of the atlanto-occipital joints. Posteriorly, it is related to the rectus capitis posterior minor muscle and, anteriorly, to the dura mater. Interdigitations with both the dura mater and the related rectus capitis posterior minor muscle parenchyma may be present in this ligament; additionally, a connective tissue bridge (exhibiting increased vascularity) joining the rectus capitis posterior minor muscle to the spinal dura is frequently present, particularly in the midline [14, 26–28]. This myoligamentous complex (comprising the posterior atlanto-occipital membrane, interspinous ligament, ligamentum nuchae, rectus posterior major and minor muscles and obliquus capitis supripr and inferior muscles) adds further stability to the craniocervical junction [29]. An important consideration in trauma of this component of the craniocervical junction is the vertebral artery which pierces the posterior atlanto-occipital membrane and then the dura mater before entering the posterior fossa.

### Nuchal ligament (ligamentum nuchae)

This is a cephalic extension of the supraspinous ligament and extends from the spinous process of the C7 vertebra attaching to the inion of the occipital bone. A sturdy structure, it limits hyperflexion of the cervical spine [14].

There are a number of other anatomically defined small “accessory” ligaments related to the craniocervical junction in addition to the more substantial ligaments outlined above (Table 1). While demonstrable on cadaveric dissection, both the identification of these smaller ligaments (or need to identify these ligaments) on clinical MRI assessment as well as the contribution of these structures to overall craniocervical junction stability is limited and, as such, injury to these ligaments in isolation is probably of low clinical significance. However, individual ligamentous injury at the craniocervical junction infrequently occurs in isolation and, therefore, as an example, MRI evidence of injury to the apical ligament, which is an inconsistent ligament on normal cadaveric dissection and contributes little to craniocervical stability, is still a valuable MRI finding when present as it indicates the likelihood of other coexisting more clinically relevant osseo-ligamentous injuries which should then be sought on the MRI sequences available.

**Table 1:** Anatomically discernible ligaments related to the craniocervical junction and MRI discernibility as discrete ligamentous structures, and relative contribution to stability of the craniocervical junction (in isolation)

Anatomical ligaments of the craniocervical junction	MRI-discernible	Stability contribution
Tectorial membrane	+	++
Alar ligament	+	++
Cruciform (cruciate) ligament - transverse	+	++
Cruciform (cruciate) ligament - longitudinal	+/−	+/−
Apical ligament	+/−	+/−
Barkow ligament	+/−	+
Atlanto-occipital capsular ligament	+	+
Atlanto-axial capsular ligament	+	+
Anterior atlanto-occipital membrane	+	+
Posterior atlanto-occipital membrane	+	+
Anterior atlanto-axial membrane	+	+
Nuchal ligament (ligamentum nuchae)	+	+
Lateral atlanto-occipital ligament	−	−
Accessory atlanto-axial ligament	−	−
Transverse occipital ligament	−	−

## Normal variant mimicking fracture

There are a number of developmental anomalies of the craniocervical junction which, while uncommon, can mimic fractures of the craniocervical junction but should not be misinterpreted as such as this can have significant ramifications on the management of patients presenting for imaging with a question of craniocervical injury.

### Condylus tertius

**Fig. 5:**
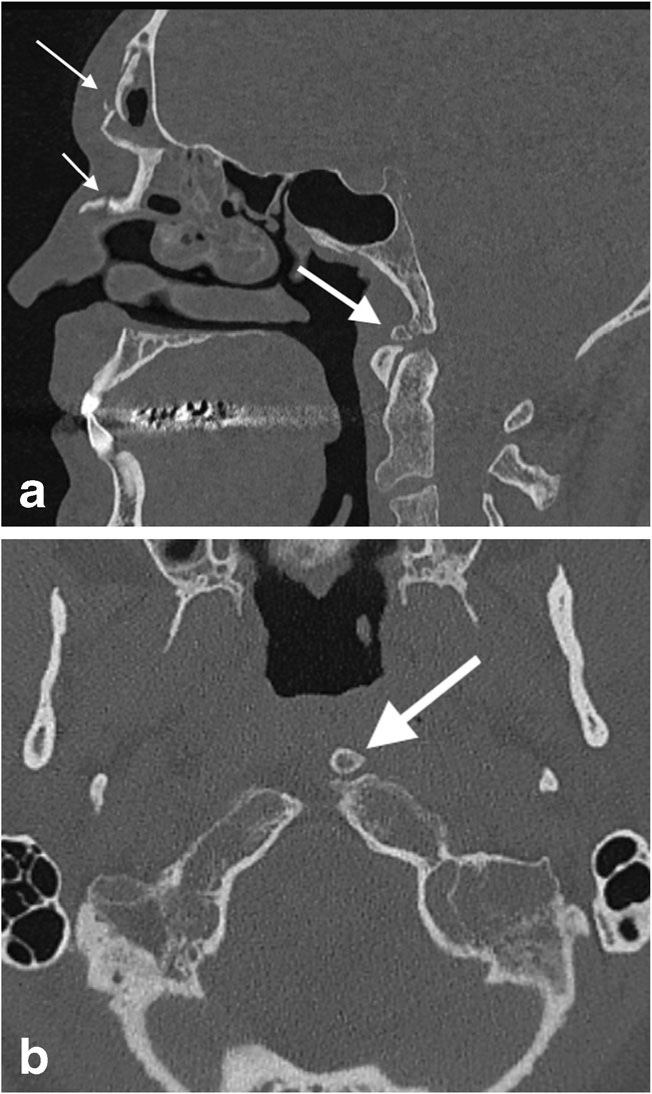
Condylus tertius: **a** sagittal reformatted CT image through the craniocervical junction of a 54-year-old male cyclist who fell of his bicycle at speed sustaining facial fractures. The condylus tertius of unfused hypochordal bow is demonstrated (*thick arrow*) and is an important mimic of fracture at the basion. Note the depressed comminuted facial fractures involving the frontal sinus and nasal bone (*thin arrow*s); **b** axial CT image of the same patient demonstrates the unfused bone remnant at the left side of the basion (*arrow*), not to be mistaken for a displaced fracture fragment

When the hypochordal bow of the fourth occipital sclerotome (proatlas) fails to integrate, an ossified remnant may be evident at the caudal end of the basi-occipit called the condylus tertius or the third occipital condyle (Fig. 5a and b). It is usually single but may be multiple and may form an arthrosis or pseudoarthrosis with the odontoid process or the anterior arch of the atlas. A well-corticated margin and a site typical of the embryological location of the hypochordal bow as well as the occasional association with an os odontoideum (see below) will aid radiological distinction from fracture [30].

### Posterior rachischisis

**Fig. 6:**
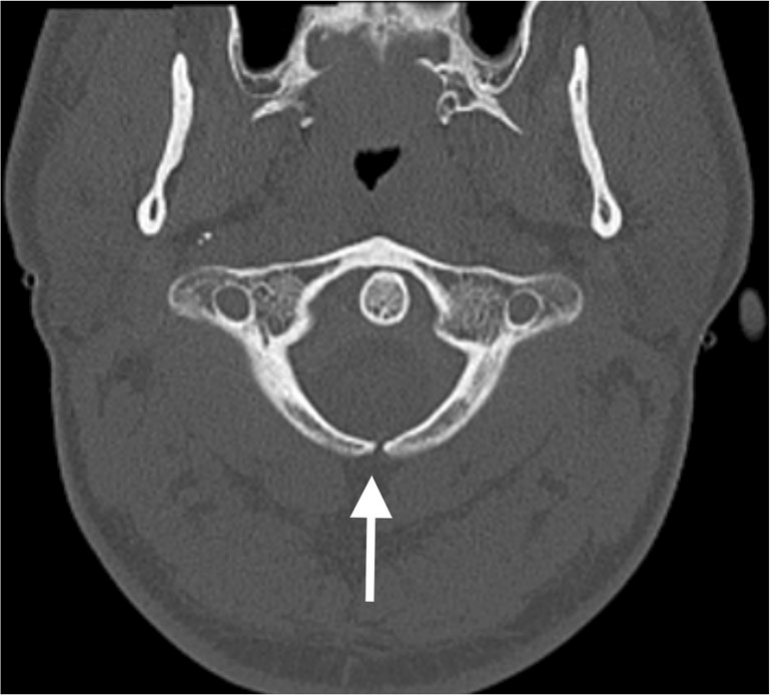
Posterior rachischisis: axial CT image through the atlas (C1 vertebra) of the craniocervical junction of a road traffic accident victim (pedestrian versus car with “bullseye” impact of the victim’s head against the windscreen of the car) demonstrating a developmental posterior rachischisis (*arrow*) which should not be confused with an acute fracture. The well-corticated margin of the cleft should alert one to the developmental nature of the anomaly in addition to the typical location and unity of the defect compared to a traumatic aetiology

**Fig. 7:**
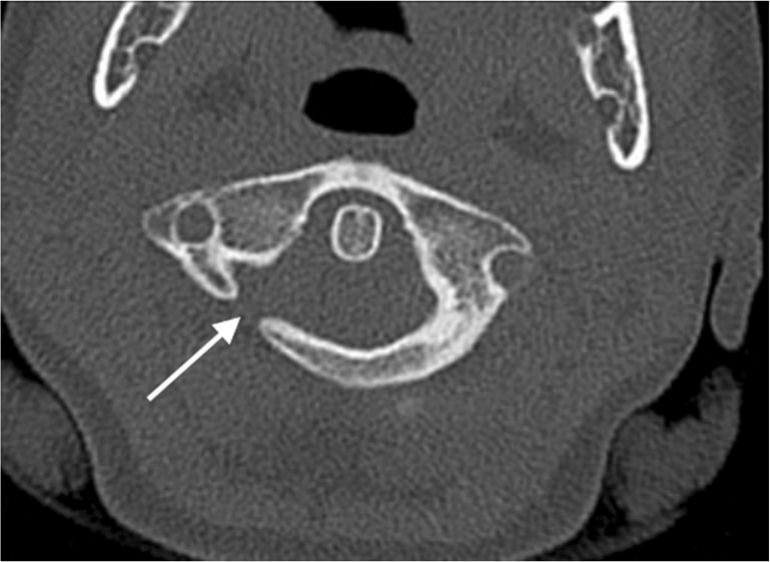
Unilateral posterolateral rachischisis: axial CT image through the atlas (C1 vertebra) of the craniocervical junction of an 8-year-old female child involved in a road traffic accident (pedestrian versus car) who sustained head injuries with seizures. A developmental unilateral right posterolateral rachischisis is present (*arrow*). There were no craniocervical junction or sub-axial cervical spine fractures present

**Fig. 8:**
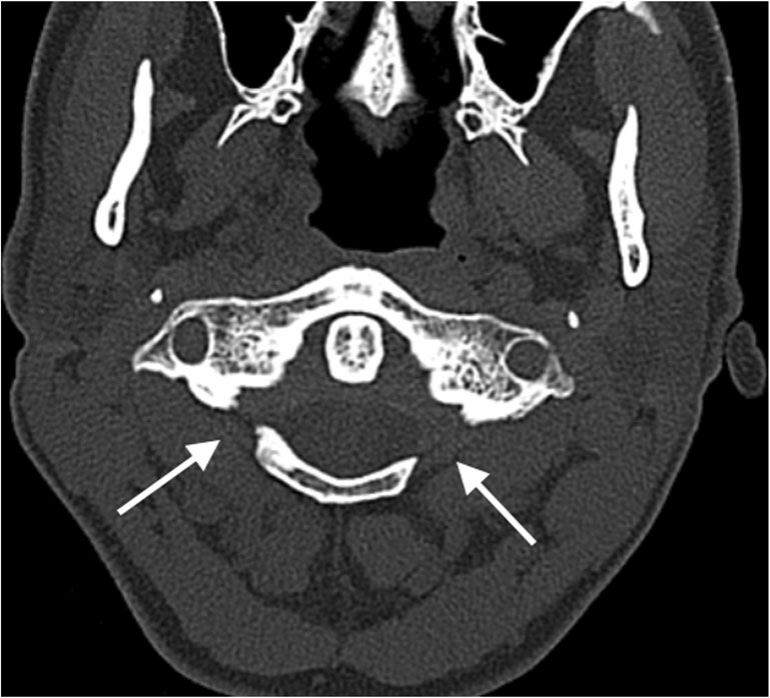
Bilateral posterolateral rachischisis: axial CT image through the atlas (C1 vertebra) of the craniocervical junction of a 47-year-old female who suffered blunt trauma to the head and complained of neck pain. Bilateral developmental posterolateral rachischisis is evident (*arrows*). There was no acute fracture of the craniocervical junction present

Absence of the posterior arch of the atlas is rare and usually isolated but can be associated with bilateral atlanto-axial subluxation or “offset”, mimicking Jefferson’s fracture [30]. Developmental clefts of the arch of the atlas are more common. Such rachischisis is more common posteriorly [30, 31]; the vast majority are midline (97 %; Fig. 6). Less commonly, a (postero)lateral rachischisis may be observed through the region of the sulcus of the vertebral artery (3 %); this may be unilateral (Fig. 7) or bilateral (Fig. 8).

### Anterior rachischisis/split atlas

**Fig. 9:**
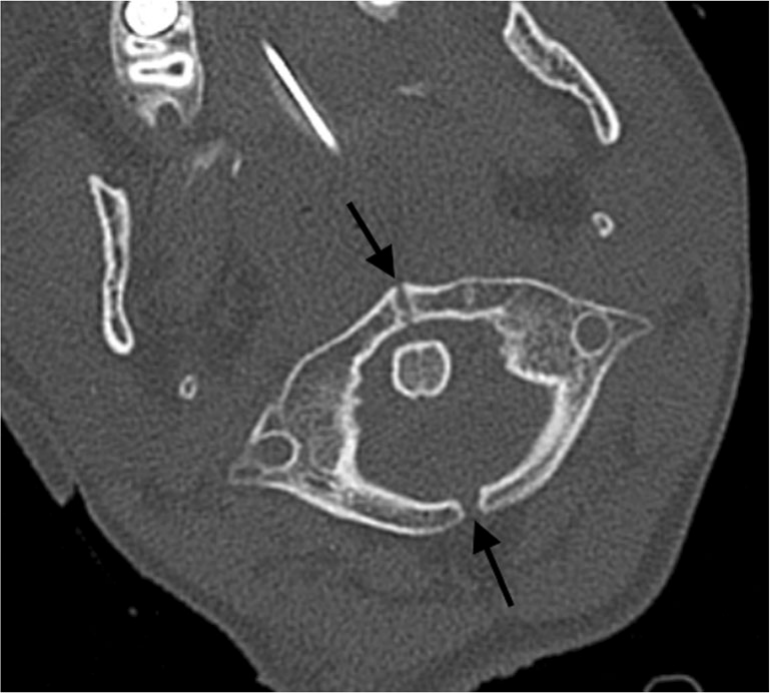
Split atlas: axial CT image through the atlas (C1 vertebra) of the craniocervical junction of a 9-year-old female struck by a car and sustaining severe chest injuries. The victim was intubated at the roadside. A developmental “split atlas” is present with anterior and posterior rachischisis defects present (*arrows*)—this is an important but rare mimic of fracture of the C1 vertebra. There were no acute fractures of the craniocervical junction in this patient

Anterior arch rachischisis is rare (occurring in less than 0.1 % of autopsy dissections) and is typically associated with a posterior arch rachischisis in which case it may be termed a “split atlas” (Fig. 9) [30–32].

### Ossiculum terminale

The persistent ossiculum terminale results from failure of fusion of the secondary ossification centre (“terminal ossicle”) to the remainder of the odontoid process which has usually occurred by 12 years of age. It may be confused with a (type I) odontoid fracture. Identification of a smooth corticated margin is, again, central to discriminating the two aetiologies [5–11, 30, 32].

### Os odontoideum

**Fig. 10:**
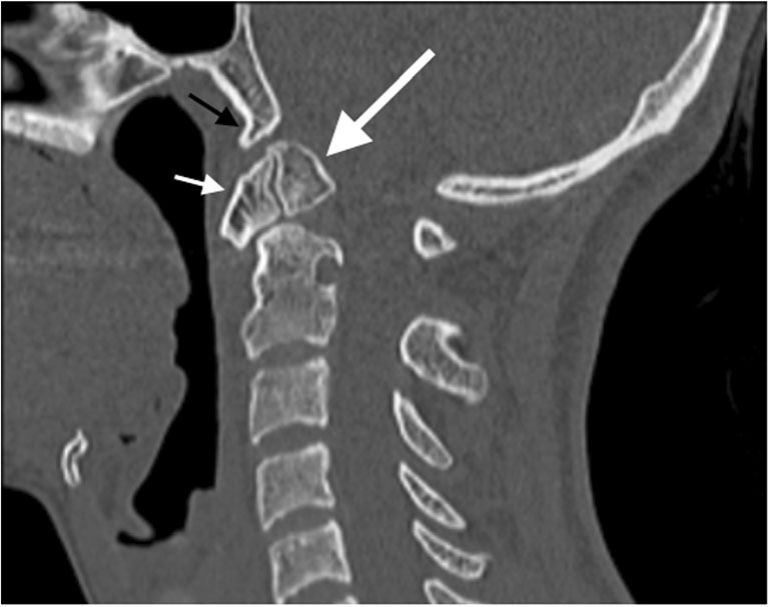
Os odontoideum: sagittal reformatted CT image of the craniocervical junction performed as part of a trauma CT assessment of a young female victim of a fall from height demonstrates a pre-existing os odontoideum (*large white arrow*) mimicking a type II odontoid process fracture. While the (type II) odontoid fracture is typically associated with a flattened uncorticated sharp margin to the adjacent body of the axis and a normal morphology to the anterior arch of the atlas, the os odontoideum exhibits a well-corticated convex upper margin. A commonly associated rounded hypertrophic anterior atlas arch is also present (*small white arrow*). This, together with an associated condylus tertius (*small black arrow*) which represents a remnant hypochordal bow, further confirms the non-acute traumatic aetiology of the odontoid process appearances

First described in 1886 by Giacomini and derived from the Latin *os* meaning *bone* and *odontoideum* meaning *tooth-form*, this represents a separate ossicle with a smooth cortical border lying superior to a small hypoplastic dens and body of the axis in the location of the odontoid process (Fig. 10) and may simulate a (type II) odontoid fracture [5–11, 30, 32]. It still remains contentious if the os odontoideum represents a post-traumatic acquired anomaly or a truly congenital anomaly. The anterior arch of the atlas may be rounded and hypertrophic in contrast to the normal anterior arch morphology. While the (type II) odontoid fracture is typically associated with a flattened uncorticated sharp margin to the adjacent body of the axis and normal morphology to the anterior arch of the atlas, the os odontoideum exhibits a well-corticated convex upper margin and rounded hypertrophic anterior atlas arch (Fig. 10).

### Calcification in the alar ligament

**Fig. 11:**
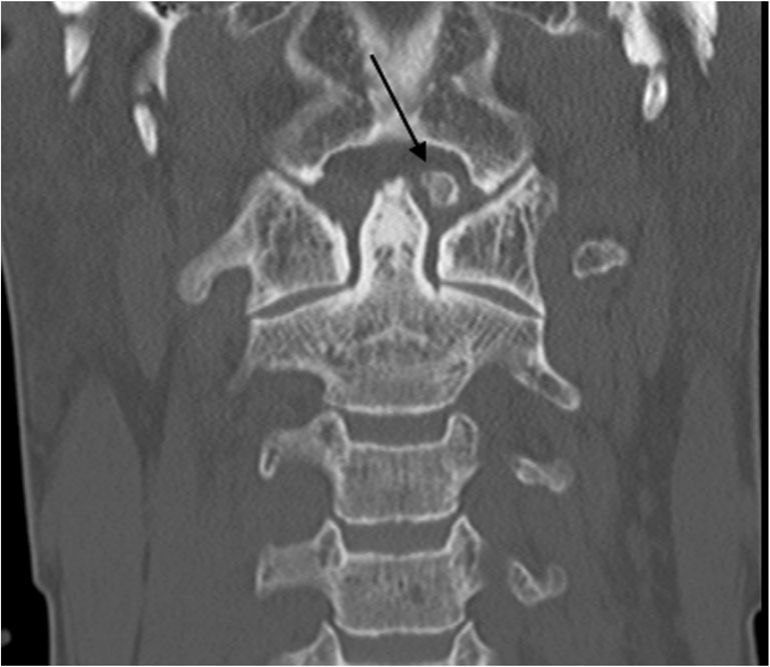
Calcification of the alar ligament: coronal reformatted CT image of the craniocervical junction performed as part of a trauma CT assessment of a 59-year-old male driver involved in motor vehicle collision complaining of neck pain on presentation to hospital. The well-circumscribed nature of this nodular area of calcification/ossification in the left alar ligament helps to discriminate this lesion from a fracture

Rarely, nodular calcification/ossification can be seen in the alar ligament which can mimic type III fracture of the occipital condyle or type I fracture of the odontoid process (see below). While rare, this can occasionally present as a imaging diagnostic dilemma in the unconscious or intubated and ventilated polytrauma patient with high-risk injuries for craniocervical trauma. The calcification usually presents as nodular relatively well-circumscribed calcification/ossification in the region of the alar ligament on CT imaging (Fig. 11) [33].

## Craniocervical junction blunt traumatic injury

### Basiocciput fractures

These fractures account for only 2 % of cranial fractures but the associated mortality is high, estimated at between 24 % to 80 % because of the proximity to the brainstem and the high incidence of neurological injury (particularly cranial nerve VI) and vascular injury [34]. The characteristic patterns described are transverse, oblique and longitudinal. The transverse and oblique patterns typically result from lateral blunt force impact or crush injuries, and associated cranial nerve injury and internal carotid injury has been described. The longitudinal fractures result typically from an axial loading mechanism through the vertex and may be associated with vertebrobasilar vascular injury and brainstem infarction.

**Fig. 12:**
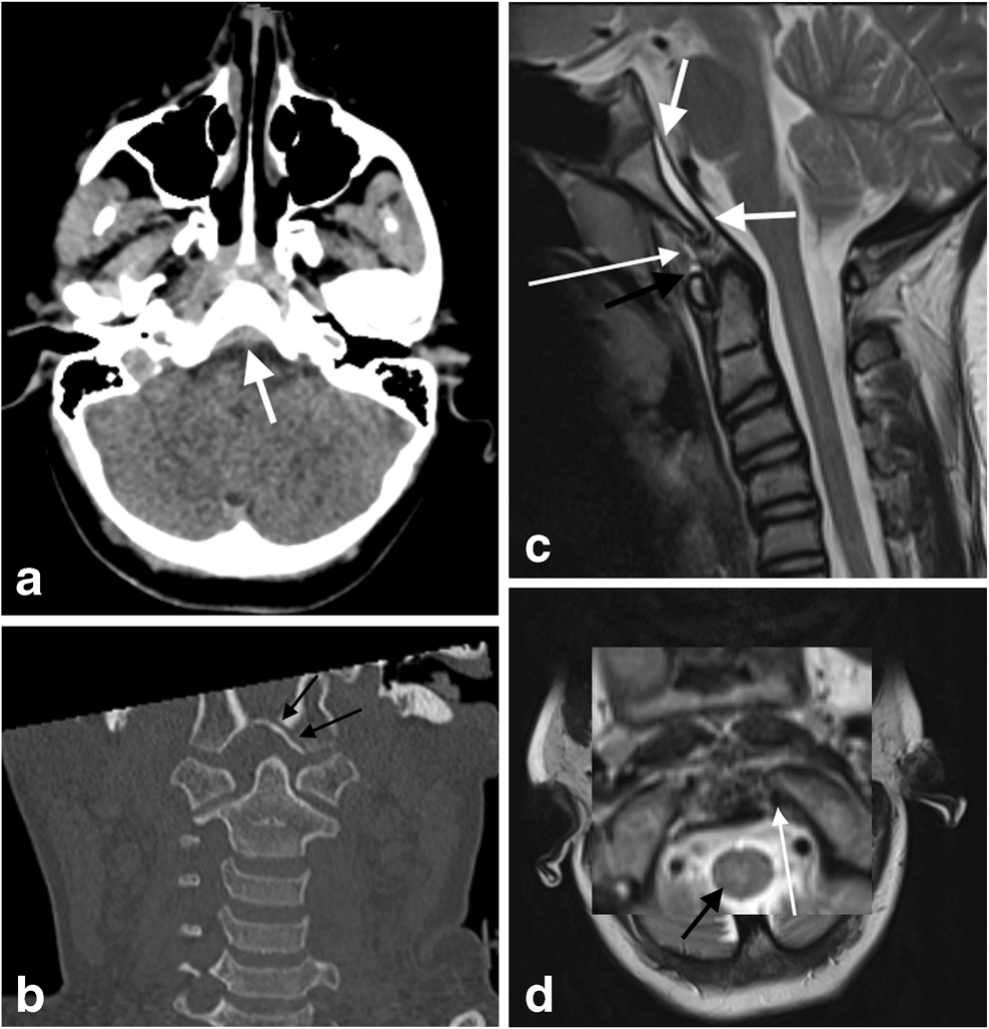
Retroclival epidural haematoma in a 12-year-old female who was a pedestrian struck by a car travelling at high speed: **a** axial non-contrast CT brain image through the posterior fossa demonstrates hyperdense retroclival abnormality (*white arrow*) in keeping with epidural haematoma; **b**: coronal reformatted CT image of the craniocervical junction of the same patient demonstrates a left basiocciput fracture extending to the left occipital condyle (type II occipital condyle fracture pattern; *black arrows*). There is asymmetrical widening of the right lateral atlanto-dental space raising the likelihood of right alar ligament disruption; **c**: sagittal T2-weighted image of the same patient through the craniocervical junction demonstrates the retroclival epidural haematoma (*short white arrows*) associated with traumatic signal change oedema and haemorrhagic fluid tracking between the deep and superficial layers of the anterior atlanto-occipital membrane (*thin long white arrow*) and around the membrane over the superior aspect of the anterior arch of the C1 vertebra (atlas; *black arrow*); **d**: axial T2-weighted image through the craniocervical junction at the level of the occipital condyles of the same patient with magnified inset demonstrating an intact left alar ligament (*white arrow*) but loss of integrity of the right alar ligament in the comparative contralateral location as a result of rupture. A traumatic oedematous contusion signal abnormality is present in the right and central cervico-medullary parenchyma (*black arrow*)

Basiocciput fractures may be seen in conjunction with craniocervical injury including the rare entity of the retroclival epidural haematoma (Fig. 12a–d). The retroclival epidural haematoma seems to be more common in the paediatric population; the reduced stability of the paediatric craniocervical junction because of smaller occipital condyles and a more horizontally orientated atlanto-occipital articulation presumably predisposes the infant craniocervical junction to this injury. The relatively reduced adherence and strength of the paediatric tectorial membrane may allow traumatic detachment and disruption of local vascular structures such as the basilar venous plexus, the dorsal meningeal branch of the meningohypophyseal trunk and a meningeal branch of the ascending pharyngeal artery (which anastomoses with the meningohypohyseal trunk, inferolateral trunk and arterial arcades related to the odontoid process) leading to blood accumulation in the retroclival area. Regardless, in the adult and paediatric populations, the retroclival epidural haematoma indicates traumatic injury of the sturdy tectorial membrane and suggests a traction/distraction injury mechanism [18, 34].

### Occipital condyle fractures

Until the emergence of CT technology, occipital condyle fractures were considered rare but, in fact, were probably significantly under-reported. The first report of an occipital condyle fracture may date back to 1817 when Sir Charles Bell described the case of a patient who was well until the time of discharge when he reached down to pick up his belongings and died suddenly—an occipital condyle fracture was identified at post-mortem and was presumed to have compressed the medulla [35, 36]. The reported incidence of occipital condyle fractures ranges from 4 % to 19 % [35, 37–40].

On the medial aspect of the occipital condyle, there is a tubercle for attachment of the alar ligament. The hypoglossal canal is an important anatomical relation located above the middle third of the occipital condyle and transmits the hypoglossal nerve (cranial nerve XII), a meningeal branch of the ascending pharyngeal artery and an emissary vein. An important lateral relation to the occipital condyle is the jugular foramen which transmits cranial nerves IX–XI, the internal jugular vein, the inferior petrosal sinus and the posterior meningeal artery [41–44].

Clinical presentation of occipital condyle fractures is variable but concomitant head injury is a frequent finding. The anatomical location of the occipital condyles means that the brainstem, the lower cranial nerves (cranial nerves IX–XII) and venous and arterial vessels are at particular risk in the event of fracture. Lower cranial nerve palsies (including Collet–Sicard syndrome where all of the cranial nerves IX–XII are affected) may be acute in two-thirds of cases [35, 41, 45–51]. Delayed cranial nerve palsies may result from migration of fracture fragments or proliferation of fibrous tissue. Vascular complications related to occipital condyle fractures include internal carotid artery and vertebral artery traumatic dissection, arteriovenous fistulae of the posterior inferior cerebellar artery and Wallenberg syndrome (lateral medullary syndrome) [35, 49].

CT assessment is mandatory to establish or confirm the diagnosis. MRI allows evaluation of the associated ligaments and, in particular, integrity of the alar ligament, transverse ligament and the tectorial membrane; it also allows evaluation of the relationship of the fractured segment on surrounding structures, particularly the brainstem and neurovascular structures.

**Fig. 13:**
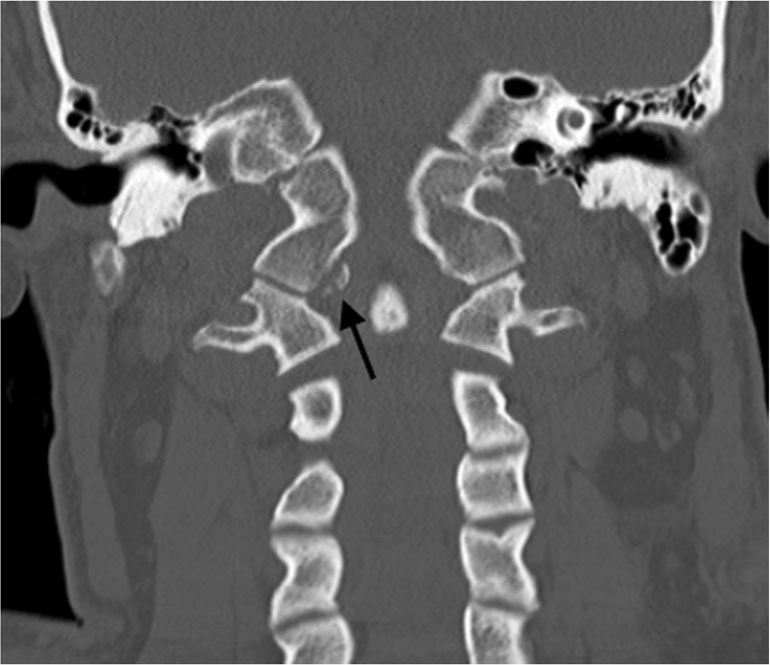
Type I occipital condyle fracture: coronal reformatted CT image through the craniocervical junction performed as part of a CT trauma assessment in a middle-aged male driver in a high-speed motor vehicle collision. A comminuted right occipital condyle fracture is present (*arrow*). The victim also sustained severe chest trauma and succumbed to his traumatic injuries the same day

**Fig. 14:**
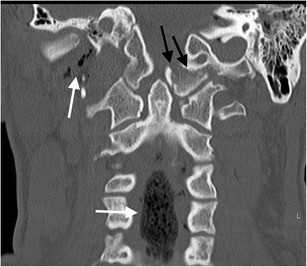
Type II occipital condyle fracture: coronal reformatted CT image of the craniocervical junction performed as part of a CT traumogram in a young male pedestrian struck by a bus. The fracture of the left occipital condyle is associated with extension into the right basiocciput (*black arrows*). Note the associated soft tissue emphysema and pneumorachis within the anterior epidural space of the cervical spine *(white arrows*)

**Fig. 15:**
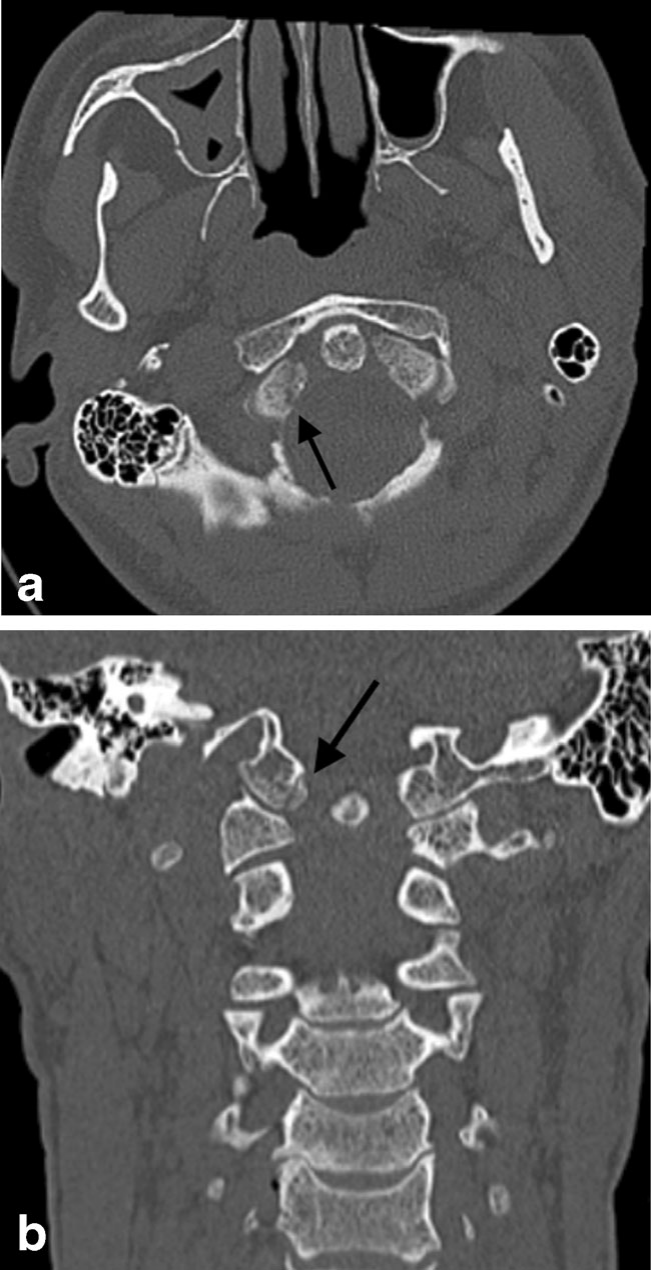
Type III occipital condyle fracture: **a** axial CT image through the atlanto-occipital level performed as part of a polytrauma CT assessment of a young female pedestrian struck by a motorcyclist travelling at speed; **b** coronal CT image of the same patient. The type III occipital condyle fracture can be subtle on CT imaging [indicated here on the right (*arrow*)] but may indicate a high-energy traumatic mechanism to the craniocervical junction with potentially unstable craniocervical spine injury related to alar ligament avulsion

The most widely accepted classification system of occipital condyle fractures was described by Anderson and Montesano and incorporates the probable mechanism of injury and the potential risk of resultant instability [52]. This system describes three types of occipital condyle fractures. Type I is an impaction type fracture resulting in comminution of the occipital condyle with or without minimal fragment displacement and the mechanism is believed to be axial loading similar to Jefferson’s fracture; this fracture type is considered stable because the tectorial membrane and contralateral alar ligament are intact (Fig. 13). However, bilateral lesions may clearly be unstable. Type II fracture (Fig. 14) is part of a more extensive basioccipital fracture involving one or both occipital condyles and is associated with intact tectorial membrane and alar ligaments, preserving stability. The type III fracture (Fig. 15) is an avulsion fracture resulting in medial fragment displacement into the foramen magnum; in this fracture type, the contralateral alar ligament and tectorial membrane may have been stressed resulting in partial tear or complete disruption and it is thus considered potentially unstable.

### Atlanto-occipital (occipito-atlantal) dislocation

This injury is associated with both high mortality and significant neurological morbidity: the force required to cause atlanto-occipital dislocation is such that the injury often proves fatal and, therefore, antemortem imaging of this traumatic injury is uncommon [53–58]. Under normal anatomical conditions, the convex occipital condyles sit within the concavity of the lateral masses of the atlas. The joint is surrounded by a loose capsule. In the paediatric population, the articulating surface of the atlas at this joint is less concave, probably contributing to the greater incidence of atlanto-occipital dislocation in this group [57, 58]. The most important ligaments for stability of the atlanto-occipital articulation are the cruciform and alar ligaments and the tectorial membrane. These ligaments are underdeveloped in the paediatric population, further contributing to the incidence of this injury in this patient group.

**Fig. 16:**
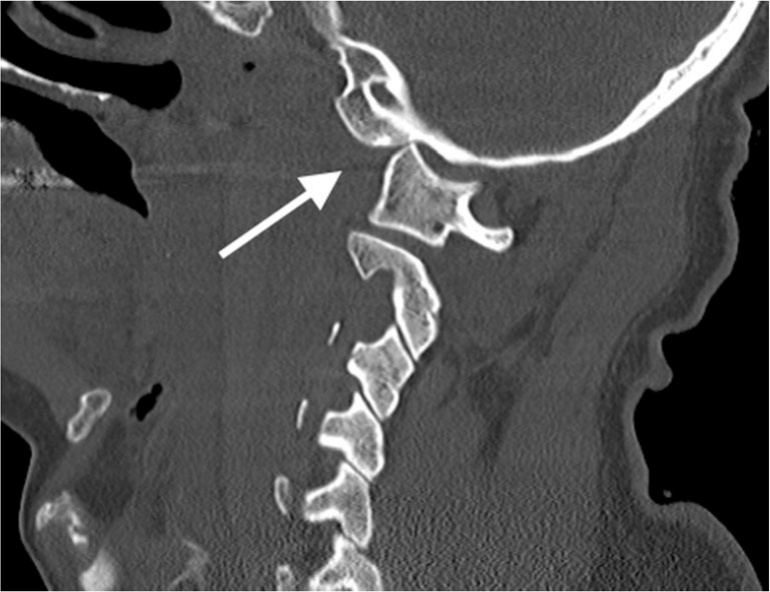
Anterior atlanto-occipital dislocation (type I dislocation): sagittal (right) reformatted CT image of craniocervical junction of a post-mortem CT study of a victim of young male patient who sustained high--force impact to the cranium as a result of an industrial accident with resultant bilateral atlanto-occipital dislocation (right joint dislocation indicated by *arrow*). The victim died at the scene: such craniocervical trauma carries a very high mortality and, as a result, ante-mortem imaging of such injuries is uncommon

**Fig. 17:**
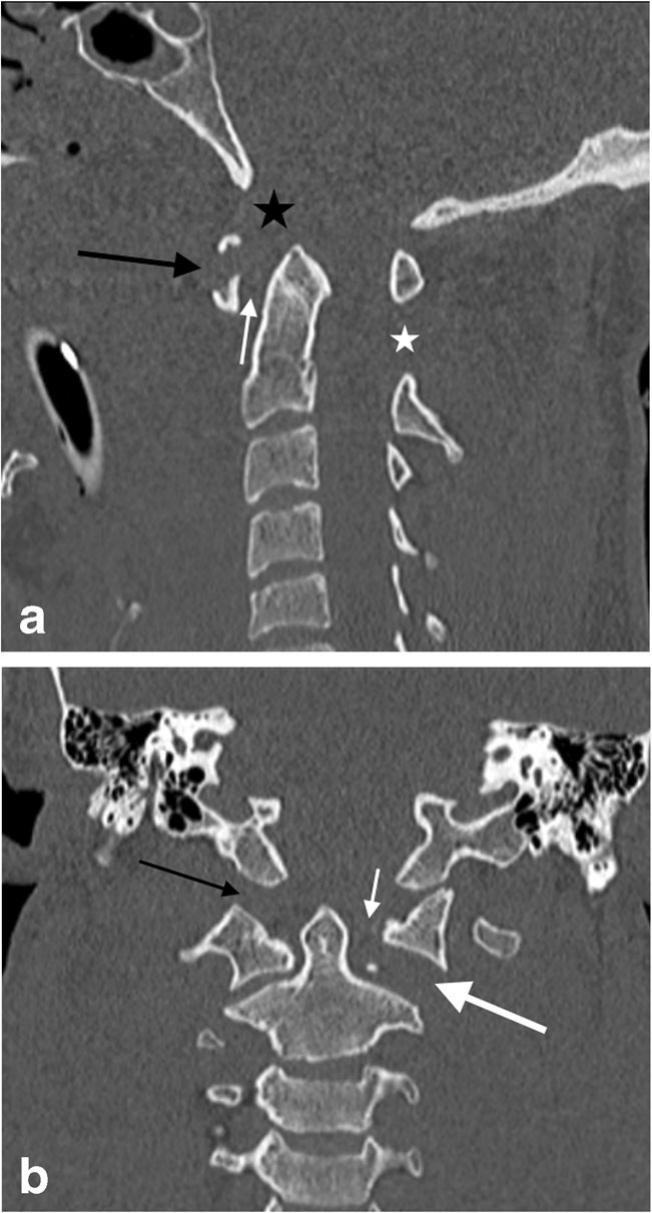
Jefferson’s fracture in a young male victim of a motor vehicle accident who suffered cardiac arrest at the scene and succumbed to his injuries shortly after presenting to the trauma centre: **a** sagittal reformatted CT image of the craniocervical imaging assessment demonstrates severe craniocervical junction disruption with Jefferson’s fracture (type V) of the atlas (*black arrow*) and associated abnormally widened predental (atlantodental); *white arrow*) in keeping with transverse ligament disruption and a pathologically widened basion-dens interval (*black asterisk*) in keeping with a distraction mechanism likely disrupting the critical alar ligaments, cruciform ligament (vertical band) and probably the tectorial membrane to some degree as well as the C1-C2 components of the ligamentum nuchae and flavum (*white asterisk*); **b** coronal reformatted CT image of the same patient confirms type II atlanto-occipital distraction (*black arrow*) associated with Jefferson’s fracture and disruption of the atlantoaxial joint on the left (*large white arrow*); the left atlantodental interval is abnormally widened (*small white arrow*) associated with Jefferson’s fracture of the atlas

The Traynelis classification is largely descriptive and divides atlanto-occipital dislocation into three types which are determined by the direction of dislocation of the occipital condyles: type I injuries represent anterior displacement of the occipital condyles in relation to the atlas (Fig. 16); type II injuries are distraction injuries with vertical displacement of the occipital condyles in relation to the atlas (Fig. 17a and b); type III injuries describe posterior displacement of the occipital condyles relative to the atlas [58–60]. Both the basion-dens interval (which is abnormal if greater than 10 mm in the adult and greater than 12 mm in the paediatric patient) and the condyle-atlas interval (which is abnormal if greater than 2 mm in the adult and greater than 5 mm in the paediatric patient) can be used to identify abnormality at the atlanto-occipital articulation; additionally, anterior displacement of the posterior margin of the odontoid peg and body of the axis relative to the basion greater than 12 mm or posterior displacement of the posterior margin of the same relative to the basion greater than 4 mm represents an abnormal relationship [58–60].

### Fractures of the C1 vertebra (atlas)

**Fig. 18:**
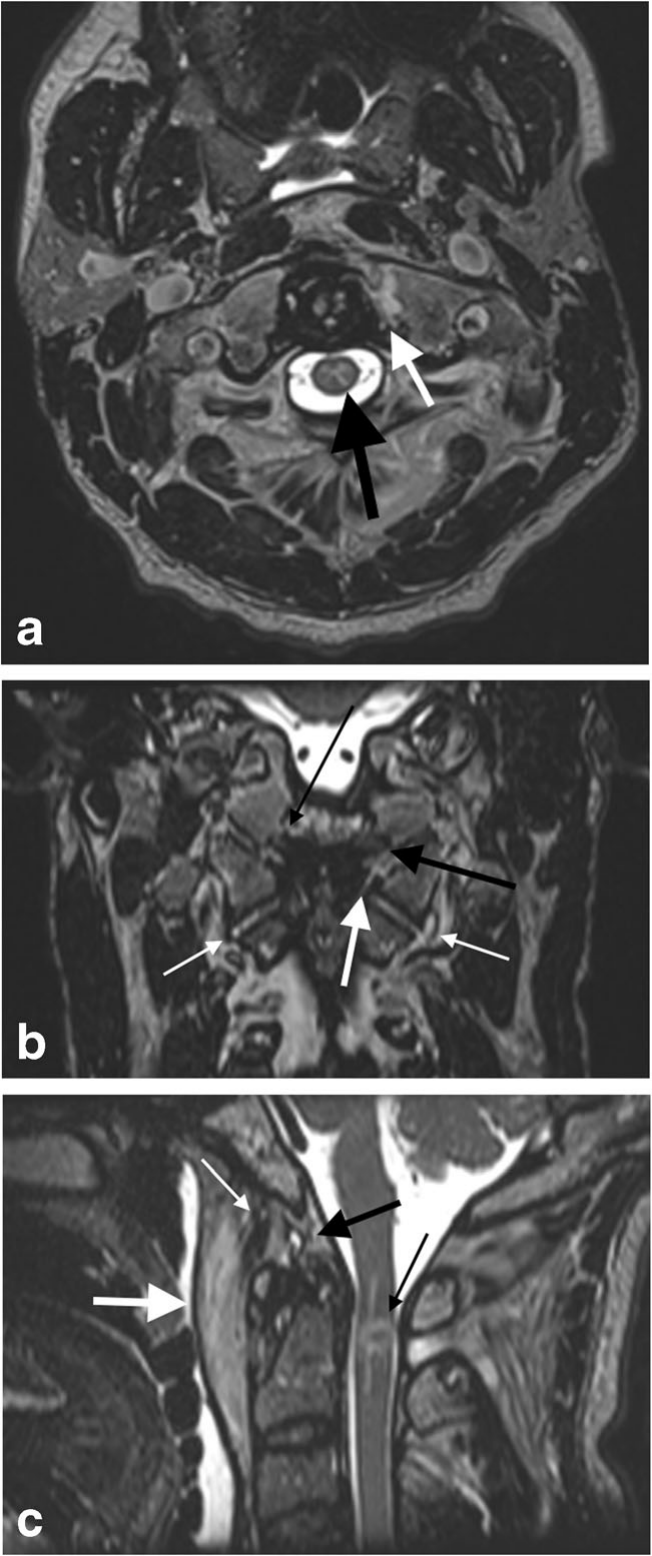
Transverse ligament and alar ligament rupture in a middle-aged male driver of a car involved in a collision with a bus; the victim was quadriparetic and respiratory-compromised at the scene: **a** axial image of 3D T2 SPACE MRI sequence demonstrates marrow oedema and fracture of the left anterior arch of the atlas extending to and involving the medial tubercle attachment of the transverse ligament on this side associated with rupture and detachment of the the transverse ligament at this site (*arrow*). Cord oedema is evident (*black arrow*); **b** coronal image of the same MRI study demonstrates traumatic signal abnormality and tear of the left alar ligament (*thick black arrow*) as well as the loss of integrity of the left side of the transverse ligament (*thick white arrow*). Some intrasubstance high-signal strain injury is noted affecting the right alar ligament at its condylar attachment (*thin black arrow*). Traumatic effusions are present in the atlanto-occipital and atlanto-axial joints bilaterally but are more marked on the left indication associated with capsular ligament strain injury (*thin white arrows*); **c** sagittal image of the same MRI study demonstrates haemorrhagic cord injury at the C1-C2 level (*thin black arrow*), abnormal haemorrhagic signal and fullness in the supra-odontoid space (“apical cave”) related to the apical ligament (*thick black arrow*), an intact tectorial membrane but abnormal traumatic signal change in the anterior atlanto-occipital membrane (*thin white arrow*) associated with a large haematoma in the prevertebral space (*thick white arrow*)

Estimated to account for 25 % of craniocervical injuries, the most common causes are motor vehicle accidents and falls [29, 61–65]. Atlas fractures can occur in isolation but are frequently associated with fractures of the axis and the subaxial cervical spine and may also be associated with rupture of the transverse ligament and closed head injury [29, 61–64]. Complications related to vertebral artery dissection injury and lower cranial nerve palsies (IX–XII) have been reported [66]. Cervico-medullary parenchymal injury occurs more frequently when fractures of the atlas coexist with axis or subaxial cervical spine injury and are typically associated with transverse ligament disruption [61, 62, 64, 67] (Fig. 18a–c).

### Jefferson’s fractures

Initially described by Sir Geoffrey Jefferson, atlas fractures can be classified as type I which are fractures involving the posterior arch alone, type II fractures which involve the anterior arch alone, type III (the classical Jefferson fracture) which are bilateral posterior arch fractures associated with a unilateral or bilateral anterior arch fracture, type IV which involve the lateral mass and type V (Fig. 17a and b) which are transverse fractures of the anterior arch [29, 68, 69]. A crucial stabiliser of the atlanto-axial articulation is the transverse ligament and the integrity of this ligament determines the stability or instability of atlas fractures.

Post-mortem studies have revealed more detail of the biomechanics of such fractures [70, 71]: atlas fractures occur primarily from an axial loading mechanism; when the axial loading occurs through the occiput, distraction of the lateral masses of the atlas occurs resulting in increased radial stress on the ring of this vertebra which subsequently “fails” or fractures at the weakest points which are the junction points between the anterior and posterior arches with the lateral masses (i.e. four in total); transverse ligament injury is common with atlas fractures but, crucially, transverse ligament injuries can occur without bone injury (Fig. 21a–c). The remaining key craniocervical ligaments are usually spared unless there are associated fractures of the occipital condyles. On plain radiographic and CT imaging, an atlanto-dental interval of greater than 3 mm in an adult and greater than 5 mm in a child, particularly in the absence of any evidence of fracture, warrants MRI assessment of the craniocervical junction as this is highly suggestive of transverse ligament disruption.

A cautionary note is the radiographic normal variant of “pseudospread” of the atlas which generally occurs in children under 7 years of age and in which the ossified lateral masses of the atlas are projected beyond the ossified articular surface of the axis (C2 vertebra) [72]. This may give the false impression of a Jefferson’s fracture and has, hence, been termed “pseudo-Jefferson’s” by some authors. It is due predominantly to the differential faster growth rate of the atlas compared to the axis in infancy with the axis growth rate eventually catching up with that of the atlas. CT evaluation will clarify this false positive radiographic impression confirming an intact atlas.

### Fractures of the C2 vertebra (axis)

The incidence of neurological deficit and acute mortality associated with fractures of the axis approach 8.5 % and 2.4 %, respectively [73–76]. In addition, the incidence of neurological deficit is significantly higher in combination atlas-axis fractures than when either of these fractures occurs in isolation [73, 77] (Fig. 18a–c).

#### Odontoid fractures

**Fig. 19:**
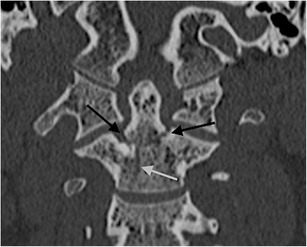
Complex type III odontoid fracture of the atlas in a 32-year-old male driver involved in a high-speed motor vehicle collision. Coronal reformatted CT image of the craniocervical junction demonstrates fracture extension into the body of the C2 vertebra (*black arrows*) with a further vertical fracture line extending through the right side of the body (*white arrow*)

The classification of odontoid fractures developed by Anderson and D’Alonzo is still in use today dividing odontoid fractures into three types [78] and with the only relevant modification introduced by Hadley et al. who defined a subclass of the type II fracture [79]. Type I is a fracture through the upper part of the odontoid process; type II is a fracture at the junction of the odontoid process with the body of the axis and is the most common type; type III is a fracture that extends downwards into the cancellous portion of the body of the axis and is the next most common pattern (Fig. 19). The type IIa subclass has additional chip fragments at the anterior or posterior aspect at the base of the dens and uniformly leads to non-union and, therefore, may warrant more pressing consideration for early surgical stabilisation and fusion [79].

The type II fracture is more prone to non-union and, therefore, such fracture types may need to proceed to surgical fusion as opposed to external/cervical collar immobilisation for halo-vest immobilisation for management [77]. The reason for this propensity to non-union seems to relate to the vascular anatomy of the axis: there are two vascular arcades that provide blood supply to the axis, one supplying the body of the axis and the other supplying the tip of the odontoid process with a resultant zone of relatively poor blood supply at the base of the odontoid process which may be rendered avascular after fracture, particularly with displacement. An additional contributory factor may be that the odontoid process is enveloped by a synovium and, as a result, lacks a periosteal blood supply. Traction by an intact apical ligament has also been suggested to cause distraction at a type II fracture site compromising endosteal healing [73].

Odontoid process fracture in the elderly population is sometimes considered a distinct entity warranting distinct management considerations, particularly type II fractures in the elderly. Lennarson et al. undertook a case–control study providing class II evidence in favour of surgery for patients over 50 years of age demonstrating a 21-times higher rate of non-union when such fractures in this age range were treated with halo immobilisation [80]

#### Hangman’s fractures

**Fig. 20:**
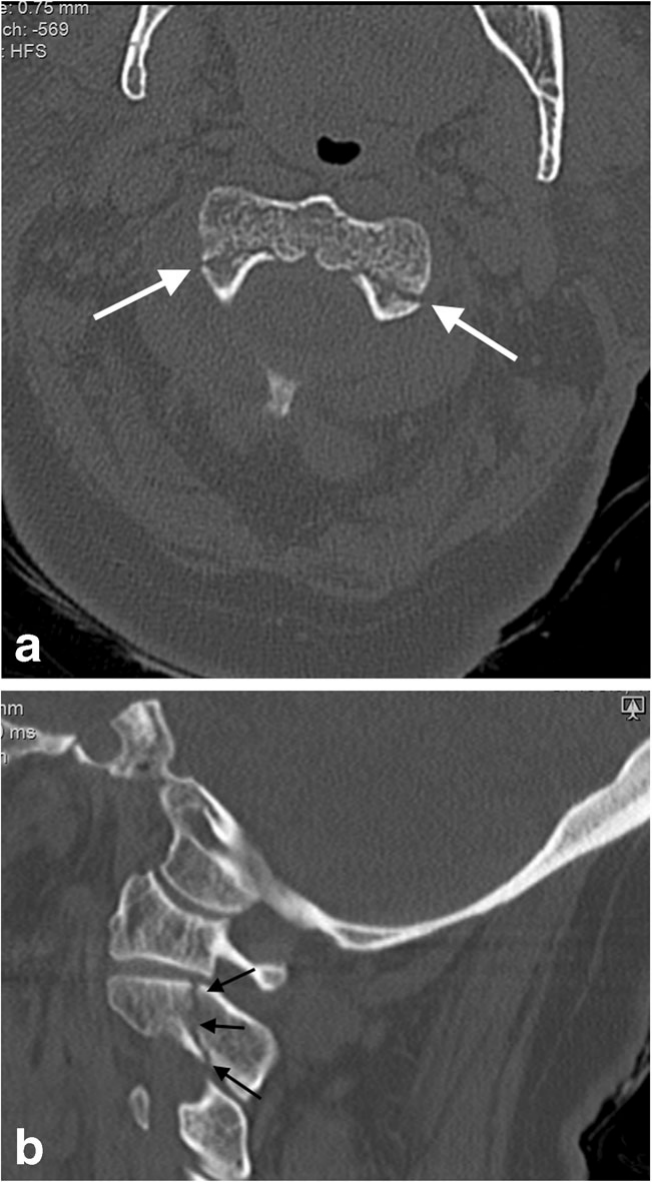
Classical hangman’s fracture in a 74-year-old male who fell down a flight of stairs: **a** axial CT image the C2 vertebra pars interarticulares demonstrating the typical fracture line locations in this type of classical hangman’s fracture (*arrows*); **b** sagittal reformatted CT image through the right pars interarticularis demonstrates the typical fracture line location (*arrows*) which was also evident in the left pars interarticularis (not shown)

Initially described in human subjects executed by hanging with the knot of the noose positioned under the submental region, this fracture-type represents bilateral fractures of the pars interarticularis of the axis (Fig. 20a and b). While similar patterns are evident in victims of motor vehicle accidents or sudden deceleration accidents and have led to common usage of the pseudonym, these traumatic spondylolistheses occur by a different biomechanical event in motor vehicle accidents or falls, i.e., hyperextension and compression as opposed to hyperextension and distraction (in judicial hanging). The incidence of spinal cord and nerve root injury as a result of hangman’s fracture is reportedly low. It is suggested that if a patient survives the initial injury, the relatively capacious canal at the level of the axis affords some protection against cord injury [73, 77, 81, 82]. The majority of these traumatic spondylolistheses can be treated with non-surgical methods such as halo immobilisation or cervical collar immobilisation.

### Ligamentous injury in the absence of fracture

**Fig. 21:**
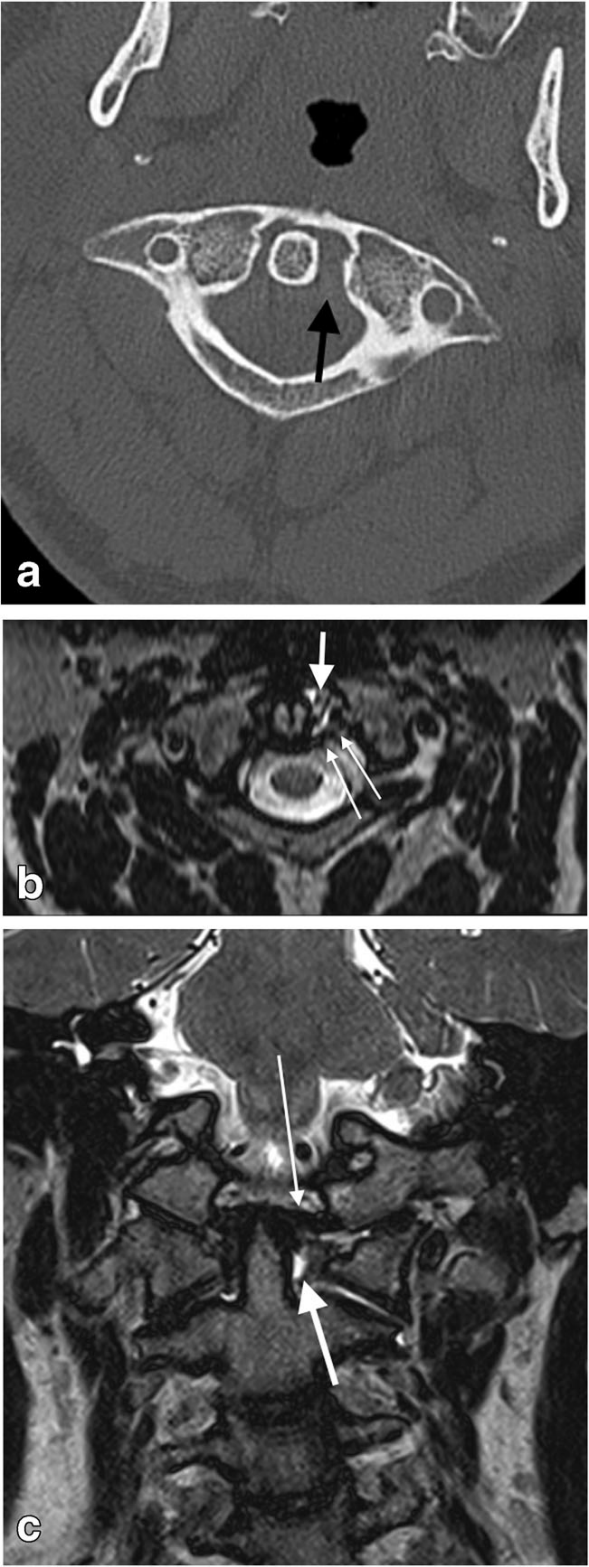
Craniocervical ligament injury in the absence of osseous injury in a 28-year-old male who sustained unimpeded fall from 3 metres associated with head injury and neck pain. There was no fracture evident on CT assessment of the craniocervical junction and sub-axial cervical spine: **a** axial CT image through the C1-C2 level of the craniocervical junction—there is widening of the left lateral atlanto-dental interval (*black arrow*) despite the absence of any fractures, raising the likelihood of craniocervical ligamentous injury; **b** reconstructed axial T2-SPACE MR image through the C1-C2 level of the same patient demonstrates disruption of the left transverse (atlantal) ligament (*thin long white arrows*) and localised haemorrhagic effusion in the widened left lateral atlanto-dental space; **c** coronal T2-SPACE MR image through the craniocervical junction of the same patient—the abnormal signal change related to the disrupted left transverse (atlantal) ligament and surrounding effusion is demonstrated (*thick white arrow*). The left alar ligament is mildly but abnormally stretched (*thin white arrow*). Small atlanto-axial and atlanto-occipital joint effusions are present (not annotated)

**Fig. 22:**
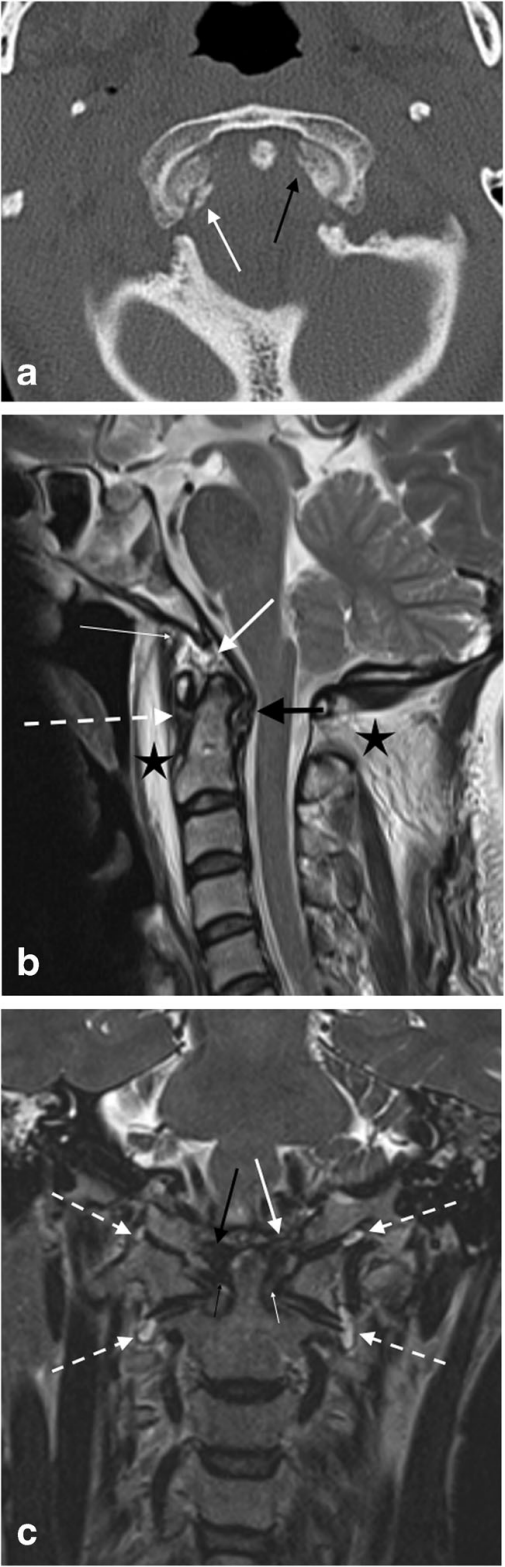
Multi-focal craniocervical junction osseo-ligamentous traumatic injury in a 51-year-old male driver of a vehicle which collided with a wall at high speed: **a**: axial CT image through the atlanto-occipital level demonstrates type —III left occipital condyle fracture (*black arrow*) and type I right occipital condyle fracture (*white arrow*); **b**: sagittal T2-weighted image through the craniocervical junction of the same patient—there is oedematous strain injury affecting the apical ligament (*bold white arrow*) with haemorrhagic fullness of the surrounding supra-odontoid space (apical cave; not annotated). There is strain injury of the transverse ligament with peri-ligamentous fluid surrounding the ligament (*black arrow*) as well as widening of the anterior atlanto-dental space (not annotated). There is partial tear of the basion attachment of the anterior atlanto-occipital membrane (*thin white arrow*) and traumatic disruption of the anterior atlanto-axial membrane (*severed white arrow*). Sizeable prevertebral haematoma and traumatic posterior paraspinal soft tissue oedema are also present (*black asterisks*); **c**: coronal image of T2-SPACE sequence through the craniocervical junction of the same patient demonstrates traumatic signal abnormality and partial tear of the left alar ligament (*long white arrow*) compared with the greater integrity of the right alar ligament (*long black arrow*), abnormal signal related to disruption of the left transverse ligament (*short thin white arrow*) compared to the integrity of the right side of the ligament (*short thin black arrow*). Traumatic effusions are evident affecting both the atlanto-axial joint capsules (Arnold’s ligaments) and the atlanto-occipital joint capsules bilaterally (*severed white arrows*). Bilateral traumatic otomastoid effusions are present (not annotated) related to lateral skull base fractures (not depicted)

Ligamentous injury at the craniocervical junction may occur in both the paediatric and adult population following trauma despite the absence of fracture of the osseous structures of the craniocervical junction (Figs. 21a–c and 22a–c). Such ligamentous injury may be acutely symptomatic and may have longer term sequelae if not identified acutely and managed appropriately, albeit frequently conservatively. There are potential medico-legal implications where failure has occurred to identify and manage such injuries expeditiously.

Traumatic alar ligament injuries typically occur near the condylar insertion. Alar ligament failure predisposes to excessive axial rotation with resultant compression of or dissection injury to the vertebral artery and damage to the spinal accessory nerves [14]. Damage to the alar ligaments is most typically secondary to high-energy blunt trauma as might occur in motor vehicle accidents. Contained alar ligament injury has also been implicated in the symptomatology related to whiplash injury [21].

MRI evidence of injury of less clinically relevant ligaments such as the apical ligament or effusion in or around the supra-odontoid space (not to be confused with normal-variant inconsistency of the apical ligament or venous plexus high signal in the supra-odontoid space, respectively) should prompt a search for strain injury, partial tear or full-thickness rupture affecting more clinically relevant stabilising ligaments of the craniocervical junction (Table 1) in spite of absence of any fracture abnormality on CT assessment.

### Imaging modalities and techniques

CT is the initial imaging modality of choice in the setting of acute traumatic injury of the craniocervical junction and cervical spine. An appropriately thin-section axial source data-set of 0.75 mm is recommended from which appropriate-resolution multi-planar reformats can be acquired (ideally, 2 mm or less). Coronal and sagittal reformats are mandatory, particularly given the availability or multi-detector CT technology and scrutiny of the axial source data-set as well as liberal use of angled/oblique reformats is also recommended. Imaging evaluation of soft tissue ligamentous injury at the craniocervical junction requires appropriate MR imaging. Inclusion of an isovolumetric T2-weighted sequence allows orthogonal post-processing assessment for enhanced radiological evaluation of the integrity of the key ligamentous structures as well as the location of any telling traumatic peri-ligamentous, intra-articular and periarticular effusions (Table 2).

**Table 2:** Royal London Hospital MRI protocol for imaging of craniocervical junction and cervical spine for ligamentous and neuroparenchymal traumatic injury evaluation (Siemens Avanto 1.5 Tesla MRI scanner)

Sequence	TR (ms)	TE (ms)	FOV (mm)	NEX (averages)	Matrix	Slice thickness (mm)
Sag T2	3500	83	240	2	269 × 384	3/3.3
Sag T1 TSE	740	11	240	3	269 × 384	3/3.3
Sag STIR	4000	30	240	2	205 × 256	3/3.3
Ax T1 TSE	610	12	200	2	190 × 320	4/4.4
Ax T2	8250	91	220	2	196 × 320	4/4.4
T2 3DSPACE COR	1500	129	160	1.6	261 × 256	0.8

*TR* time to repetition, *TE* time to echo, *FOV* field of view, *NEX* number of excitations, *Sag* sagittal, *Ax* axial, *TSE* turbo spine echo, *SPACE* sampling perfection with application-optimised contrasts using different flipangle evolutions, *COR* coronal

### Traumatic rotatory atlanto-axial subluxation

Atlanto-axial rotatory subluxation (AARS) is relatively rare in the adult population, occurring more commonly in the paediatric population. In adults, the most common cause is trauma. If the facets become locked, then the term atlanto-axial rotatory fixation (AARF) is often applied as the deformity is irreducible. Some two-thirds of the normal rotation that occurs in the cervical spine is derived from the atlanto-axial articulation. However, there is a biomechanical trade-off for this degree of mobility which is stability. It is also important to bear in mind that when the head is rotated normally, the neighbouring segments of the vertebral arteries are structurally affected: the ipsilateral vertebral artery will be kinked and the contralateral vertebral artery will be stretched. Hence, vertebral artery injury or insufficiency is a potential complication associated with AARS. Clinically, patients may demonstrate torticollis and adopt the cock-robin position of the head (because of the apparent descriptive resemblance of a robin listening for a worm in the ground) and occipital pain may occur as a result of compression of the greater occipital nerve or the C2 nerve root. Patients may also experience vertigo, nausea, tinnitus and visual disturbances, possibly related to haemodynamic compromise of the vertebral artery. Spasmodic torticollis (sternocleidomastoid muscle spasm) should be distinguished clinically from AARS—in the former, the shortened sternocleidomastoid on the side contralateral to the direction of head rotation is creating the force producing the deformity, whereas in the latter, the lengthened sternocleidomastoid muscle on the side ipsilateral to the direction of head rotation demonstrates spasm in an attempt to correct the deformity [83]. CT (with or without incorporation of a dynamic protocol in the setting of trauma if fracture is or is not demonstrated on the static CT study) and MRI (to assess soft tissue injury including ligamentous injury) are recommended. Probably the most widely used classification of AARS/AARF is that created by Fielding and Hawkins which classifies the deformity in to four types: type I is rotatory subluxation/fixation without anterior displacement of the atlas (i.e. the atlanto-dental interval is less than 3 mm); type II is rotatory subluxation/fixation with anterior displacement of the atlas of 3–5 mm; type III is rotatory subluxation/fixation with anterior displacement of the atlas greater than 5 mm; type IV is rotatory subluxation/fixation with posterior displacement [83].

### Secondary blunt traumatic vascular injury

**Fig. 23:**
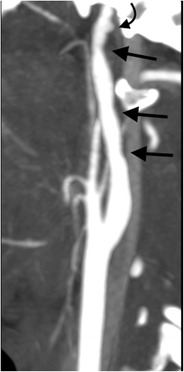
Blunt traumatic vascular dissection injury: right sagittal oblique reformatted image of CT angiography of a young female pedestrian who sustained Hangman’s fracture as a result of a “hit-and-run” motor vehicle accident demonstrates vascular dissection abnormality of the distal cervical segment of the right internal carotid artery below the skull base (*straight arrows*) with associated pseudoaneurysm formation (*curved arrow*)

The centralisation of the management of severe polytrauma into specialised trauma centres as well as utilisation of aggressive screening criteria has seen a significant rise in the incidence of documented cerebrovascular injury in blunt trauma patients, particularly when applied to patients with an injury severity score greater than or equal to 16 [84–91]. In spite of the relative infrequency of blunt carotid and vertebral artery injury, it is the potentially devastating complications of such injuries, when present, which makes pre-empting such vascular trauma an essential consideration of the radiologist. Ischaemic events do not infrequently present with some delay after a latent asymptomatic period [85, 87, 92, 93]. Traumatic vascular dissection usually begins with a traumatic intimal tear or intramural haematoma: intimal disruption results in platelet aggregation and subsequent thrombus formation which may narrow or occlude the affected vessel or embolise distally; the intimal flap may be stripped distally creating a false lumen and compromise haemodynamic stability; intramural haematoma within the media may propagate distally (cranially), narrow or occlude the vessel or expand the adventitia producing a traumatic false aneurysm (pseudoaneurysm). Traumatic arteriovenous fistulae may also occur as a consequence of such injuries. In the acute setting, CT angiography is the imaging modality of choice performed from the aortic arch through to and including the circle of Willis as vessel injury may be remote from other sites of non-vascular trauma. Miller et al. [91] spurned the Memphis criteria and Biffl et al. [87, 94] developed the Denver criteria which provide some predictive imaging risk factors for blunt traumatic carotid and vertebral artery dissection injury, of which fractures of the craniocervical junction (C0-C2) are included (Fig. 23). Early identification of blunt traumatic cerebrovascular injury allows appropriate early implementation of antithrombotic or anticoagulant therapy during the asymptomatic period post-trauma and has been shown to decrease the incidence of post-traumatic stroke and improve final neurological outcome [85, 87, 92, 93].

## Conclusion

The craniocervical junction is a unique component of the osseo-ligamentous craniospinal axis with unique embryological derivations and specific biomechanical demands. Radiologically, it warrants specific and diligent consideration in the setting of trauma. The current imaging recommendations in the trauma scenario include non-contrast CT evaluation and, where there has been a significant bony injury or there exists suspicion of ligamentous injury, MRI assessment may also be required. The proximity of the internal carotid arteries and vertebral arteries to the craniocervical junction means that a high index of suspicion for traumatic vascular dissection injury should be maintained by the radiologist where traumatic injury to this region has been sustained and CT angiography should be included in the imaging armamentarium.
